# Transcriptome Characterization of Short Distance Transport Stress in Beef Cattle Blood

**DOI:** 10.3389/fgene.2021.616388

**Published:** 2021-02-10

**Authors:** Haidong Zhao, Xiaoqin Tang, Mingli Wu, Qi Li, Xiaohua Yi, Shirong Liu, Junyi Jiang, Shuhui Wang, Xiuzhu Sun

**Affiliations:** ^1^College of Animal Science and Technology, Northwest A&F University, Yangling, China; ^2^College of Grassland Agriculture, Northwest A&F University, Yangling, China

**Keywords:** transportation stress, beef cattle, RNA-seq, hematological indices, co-expression

## Abstract

The transportation is a crucial phase in beef cattle industry, and the annual losses caused by beef cattle transport stress are substantial. Several studies have described the effect of long distance transportation stress on animal health, such as disorder in nervous, endocrine, immune, and metabolic system. However, molecular mechanisms underlying short distance transportation stress is still poorly understood. Present study aims to investigate the effect of short distance transportation by measuring the hematological indices and transcriptomic analysis. In this study, a total 10 Qinchuan cattle were used to compare the molecular characteristics of blood before and after transportation. We have found that a stress-related marker “white blood cell count (WBC)” increased significantly after transportation. The decrease in triglyceride (TG), cholestenone (CHO), high-density lipoprotein (HDL), and low-density lipoprotein (LDL) showed that energy expenditure was increased after transportation, but not enough to activate fatty decomposition. Intriguingly, the decrease of malondialdehyde (MDA) showed that cattle were more resilience to oxidative stress. The RNA-seq showed that 1,092 differentially expressed genes (DEGs) were found (329 up-regulated and 763 down-regulated) between group before and group after. The GO and KEGG enrichment showed that the metabolic pathway and B cell function related pathways were enriched. Furthermore, median absolute deviation (MAD) top 5,000 genes were used to construct a co-expression network by weighted correlation network analysis (WGCNA), and 11 independent modules were identified. Combing with protein-protein interaction (PPI) analysis, the verification of quantitative real-time PCR (qPCR) and the correlation of B cell function, *structural maintenance of chromosomes 3* (*SMC3*), *jun proto-oncogene* (*JUN*), and *C-X-C motif chemokine ligand 10* (*CXCL10*) were suggested as potential molecular markers in identification of short distance transportation. Collectively, the blood RNA-seq analysis and WGCNA indicated that the disorder of B cell differentiation, proliferation, survival, and apoptosis were the potential molecular mechanism in short distance transportation stress. In conclusion, our results provide the novel insight about potential biomarkers for short distance transportation stress, which may serve as for diagnosing and preventing this condition in beef industry.

## Introduction

Freshly cut meat has become a major product in the meat market worldwide. Livestock is bought and sold more frequently in the market economy and transportation is an integral part of the livestock industry. The key factors that can affect the welfare of animals and the economic benefits of this industry during transportation include a higher susceptibility to disease and increased disease spread (Schwartzkopf-Genswein et al., 2012; Deng et al., 2017). Transportation stress syndrome (TSS) often appears in beef cattle after ground transportation, and can affect many tissues, including the nervous, endocrine, immune, and energy supply systems (Van Engen and Coetzee, 2018).

The stressors responsible for inducing transport stress syndrome are complex, including temperature, wind, rain, hunger, thirst, crowding, shock, turbulence, social interaction, feed change, physical exertion, environmental change, and invasion of pathogenic bacteria (Delic et al., 2014). Corticosterone secretion and lipid mobilization are normal phenomena under stress conditions (Chirase et al., 2004; Kinlein et al., 2019). Although TSS gradually subside through vitamin in feed, however, in some cases, a few animals do not recover by vitamins supplement, eventually decrease the production efficiency or die (Kegley et al., 2016; Takemoto et al., 2017; Takemoto et al., 2018). Greater attention should be paid to transport stress syndrome of beef cattle (TSSBC), as their rumen digestive system needs time to recover from the dysbiosis caused by transportation stress (Deng et al., 2017; Li et al., 2019; Alfaro et al., 2020). To avoid the losses caused by TSS and better safeguard animal welfare, it is essential to provide a better condition and immune enhancing supplements for cattle during transportation. Beside extra-care it is also important to gain an in-depth understanding of the molecular mechanisms underlying TSS.

Comparing to long distance transportation stress, the molecular mechanism of short distance transportation stress is poorly understood. RNA sequencing (RNA-seq) is the method of choice for the high-throughput analysis of gene expression due to its low cost and ease-of-use, and offers a better guide for exploring the unknown information in transportation stress (Poplawski et al., 2016; Hrdlickova et al., 2017). Considering the potential application in transportation stress, blood is a suitable medium for samples collection and has low damage to cattle. This study aimed to examine the change in hematological indices and blood transcriptome after transportation, thereby providing the corresponding theoretical basis for the diagnose of TSSBC and a reference for the cost-control of beef cattle production and the safeguarding of animal welfare.

## Materials and Methods

All experimental procedures were performed in accordance with the Regulations for the Administration of Affairs Concerning Experimental Animals approved by the State Council of the People’s Republic of China. The study was approved by the Institutional Animal Care and Use Committee of Northwest A&F University (Permit Number: NWAFAC1019).

### Animal Model of Short Distance Transport Stress

Ten healthy Qinchuan beef cattle (3∼4 years old) were used in the study. The animals were kept in loose housing conditions before transportation and fed on a total mixed ration (TMR). The beef cattle were deprived of food and water until after transportation. Blood samples were collected from each animal’s jugular vein before transportation, and marked B1∼B7. Blood was collected about 17 h after last feeding. Thereafter, transportation has begun from Qinbao Cattle Industry Co., Ltd in Yangling County, Shaanxi Province to Qinbao Cattle Industry Co., Ltd in Qishan county, Shaanxi province. The average transportation density was 1.28 m^2^ a head which was similar to production process. The transportation distance was 70 km, and the vehicle was a single-layer Foton cart with a maximum speed of 60 km/h and an average speed of 30 km/h. The temperature ranged from 26 to 32°C, and the humidity was 70%. There was no other animal transported with them. After transportation, blood samples were collected again from each animal’s jugular vein immediately, and marked A1∼A7. The interval time between the two blood samples collection was 6–8 h.

### The Testing of Hematological and Biochemical Indices

To separate the serum, the blood collected in 5 mL tubes without anticoagulant, DNase and RNase and then centrifuged at 2,000 rpm for 10 min at 4°C. Blood for hematological indices detection was collected in sodium heparin anticoagulant containing tubes. Hematological indices were determined by the Aidi Pet Clinic (Yangling, China), including white blood cell count (WBC), lymphocyte number (LYMPH#), lymphocyte percentage (LYMPH%), monocyte number (MON#), monocyte percentage (MON%), red blood cell count (RBC), neutrophil granulocyte number (Gran#), neutrophil granulocyte percentage (Gran%), hemoglobin (HGB), hematocrit (HCT%), average red blood cell volume (MCV), average red blood cell hemoglobin amount (MCH), average red blood cell hemoglobin concentration (MCHC), red blood cell percentage (RDW), platelet number (PLT), average platelet volume (MPV), platelet distribution width (PDW), thrombocytocrit (PCT%). Conventional biochemical parameters were tested using a Hitachi automatic analyzer (Hitachi, Tokyo, Japan), including triglyceride (TG), cholestenone (CHO), high-density lipoprotein (HDL), low-density lipoprotein (LDL), glucose (GLU). Superoxide dismutase (SOD) level was measured using the hydroxylamine method (NJJCBIO, Nanjing, China), and malondialdehyde (MDA) content was measured using the thiobarbituric acid (TBA) method (NJJCBIO, Nanjing, China).

### RNA Isolation and Library Preparation

Blood for RNA-seq and quantitative real-time PCR (qPCR) was collected in sodium heparin anticoagulant containing tubes and stored at −80°C until further analysis. To ensure the quality of RNA in samples, the following steps were performed as quickly as possible. ACK lysis buffer lysed erythrocytes, karyocytes were harvested following centrifugation at 4,000 rpm for 6 min at 4°C, and then transfer into eppendorf tubes containing trizol, and finally stored in liquid nitrogen (Braga et al., 2017; Huang et al., 2017). Total RNA from the blood samples (*n* = 7) were isolated from leukocytes using the RNAiso Plus Kit (Takara, Tokyo, Japan). RNA concentration and quality were evaluated using Qubit2.0 fluorometer (Invitrogen, California, United States) and NanoDrop 1000 (Thermo Fisher Scientific, Massachusetts, United States). cDNA libraries were prepared using Hieff NGS^TM^ MaxUp Dual-mode mRNA Library Prep Kit for Illumina^®^ (YEASEN Biotech Co., Ltd, Shanghai, China). Libraries were quantified using Qubit2.0 Fluorometer DNA Assay Kit (YEASEN Biotech Co., Ltd, Shanghai, China) and submitted for sequencing (Illumina Xten, San Diego, United States).

### Primer Design and qPCR

qPCR was performed to verify the RNA-seq expression pattern of blood before and after transportation. β*-actin* (*ACTB*) was used as the housekeeping gene (Table 1). Reverse transcription of RNA to cDNA was performed before qPCR, carried out in the Y480 Real-Time PCR Detection System (Roche, Basel, Switzerland) utilizing SYBR green detection (Takara Bio, Tokyo, Japan). The amplification protocol was as follows: 95°C for 30 s, followed by 50 cycles of 95°C 10 s and 60°C for 30 s. Melt curve analysis was performed between 55 and 95°C, with a 0.5°C increment every 5 s. Samples were run in triplicate. All expression levels were normalized to that of *ACTB* and quantified using the 2^–^^Δ^^Δ^^Ct^ method (Livak and Schmittgen, 2001). *ACTB* was not different between animal groups before and after transportation. Twelve differentially expressed genes (DEGs) (fold-change > 2 and False discover rate (FDR) < 0.05) were randomly chosen for verification by qPCR. Hub gene (degree top 30) belonged to DEGs were also chosen for verification by qPCR.

**TABLE 1:** List of the primers used in this study.

Gene symbol	Sequence (5′–3′)		Accession
*S100A9*	F: GCTTCTCGGCTTGGTAGGAG	R: CGCCTTCTGTTTGAGCAACG	XM_005203728.2
*MS4A1*	F: GCTCCAGACCCAAAGCTAACG	R: TCAGCCACTTCAATCTGCTCA	NM_001077854.2
*RGS1*	F: CAGGCATGTTCCTCTCTGCTAA	R: CTTTCACGTCCATTCCAAAAGTC	NM_001199063.1
*CD19*	F: GCTCGAATAGGGTGGAGCAC	R: GAGGAGGCCCAGGCTGAA	XM_024984859.1
*CD14*	F: AAAGAATCCACAGTCCAGCCG	R: CCAAATAGCCCACGCTTCG	XM_005209429.4
*CEBPD*	F: CACTTTGATTCCTGCGCTCG	R: GGAGTTCGGCCTGTTTGAGA	NM_174267.2
*ATP6*	F: AATCGGAGGAGCTACACTTGC	R: AGAGCTGTTGTAGTGCTAATGCT	NC_006853.1
*CD79B*	F: GGCGGAACACACTGAAAGATG	R: CCTTGCTGTCATCCTTGTCCA	XM_005220987.4
*BANK1*	F: CCCAGTACAAGTGTTTCAACAAA	R: CCATCCGACCTCTGTCTTGT	XM_024993521.1
*CEBPB*	F: CGACCTCTTCTCCGACGACT	R: CAGACTCACGTAGCCGTACTC	NM_176788.1
*BIRC3*	F: CAAGTGGTTTCCACGGTGTG	R: GGGTAACTGGCTTGAACTCG	XM_024975220.1
*BPI*	F: GCTCCTGGAACTGAAGCACT	R: GCAGCGACTCAACCGAGAAG	XM_025000258.1
*RBM25*	F:CCGGGCACTAGGAAGACCTC	R:GATTCCAAAGCGCCTACTCG	XM_002690998.6
*CD40*	F: GTGACCCACTGACTCAGTATG	R: GCCAACTGCAACAACACATGC	XM_005214504.4
*SMC3*	F:GAAGAGTATGGAGCGCTGGAA	R:GCAGCATGCCTTGTCGATTT	NM_174295.1
*BRIX1*	F:AAGATGGCAGCGACCAAGAG	R:ATACGTCGTTCGCCTTAGCC	NM_001034529.2
*SMC5*	F: AGAGAAGGAGAAGAGGAGTGT	R: TCATATTTATCGTGAGCATTATGGG	XM_005210026.4
*JUN*	F: TGCAAACGTTTTGAGGCGAG	R: GGGCTTTAGTCCTCGGACAC	NM_001077827.1
*CXCL10*	F: CTCGAACACGGAAAGAGGCA	R: TCCACGGACAATTAGGGCTT	NM_001046551.2
*CCL5*	F: CACCCACGTCCAGGAGTATT	R: CTCGCACCCACTTCTTCTCT	NM_175827.2
*ACTB*	F: CATCCTGACCCTCAAGTA	R: CTCGTTGTAGAAGGTGTG	NM_173979.3

*S100A9, S100 calcium binding protein A9; MS4A1, membrane spanning 4-domains A1; RGS1, regulator of G protein signaling 1; CD19, CD19 molecule; CD14, CD14 molecule; CEBPD, CCAAT enhancer binding protein delta; ATP6, ATP synthase F0 subunit 6; CD79B, CD79b molecule; BANK1, B cell scaffold protein with ankyrin repeats 1; CEBPB, CCAAT enhancer binding protein beta; BIRC3, baculoviral IAP repeat containing 3; BPI, bactericidal permeability increasing protein; RBM25, RNA binding motif protein 25, CD40, CD40 molecule, SMC3, structural maintenance of chromosomes 3, BRIX1, biogenesis of ribosomes BRX1, SMC5, structural maintenance of chromosomes 5, JUN, Jun proto-oncogene, CXCL10, C-X-C motif chemokine ligand 10; CCL5, C-C motif chemokine ligand 5; ACTB, actin beta.*

### RNA-Seq Analysis and Statistics

Fast QC^1^ was used for quality control (Brown et al., 2017). Trimmomatic^2^ was used to remove reads containing adapter, reads containing poly-N, and low-quality reads (Bolger et al., 2014). Q20 was the filter standard for the following analysis. Available data was mapped to the reference genome sequence from NCBI database (Bos taurus ARS-UCD1.2) using HISAT2 tools^3^ (Kim et al., 2019). DEGs were determined by DESeq2 (Anders and Huber, 2010), and satisfy the fold change > 2 and FDR < 0.05. Gene ontology (GO) enrichment analysis and Kyoto encyclopedia of genes and genomes (KEGG)^4^ pathway enrichment analysis were performed using DAVID^5^, and satisfy the condition that FDR < 0.05 (Jiao et al., 2012). Paired *T*-test was performed in hematological and biochemical indices analysis, qPCR verification of DEGs and hub genes by SPSS 18.0 (IBM, New York, United States). For weighted correlation network analysis (WGCNA) (Langfelder and Horvath, 2008), more than 90% of the genes with counts less than 10 were removed, and median absolute deviation (MAD) top 5,000 genes were extracted to construct a co-expression network. WGCNA parameters were as follows: power = 8, minModuleSize = 30, networkType = “signed,” corType = “pearson,” TOMType = “signed,” mergeCutHeight = 0.25. The functional enrichment analysis of genes in each module was performed by DAVID dataset^5^ (Jiao et al., 2012). The protein-protein interaction (PPI) network of the interested module was constructed using the STRING online database^6^ (Szklarczyk et al., 2019). The degree algorithm of Cytohubba plugin based on Cytoscape was used to identify the high degree genes, which play a critical role in the PPI (Doncheva et al., 2019). Then degree top 30 genes were classified as hub genes.

## Results

### The Effects of Transportation Stress on Hematological and Biochemical Indices

**FIGURE 1:**
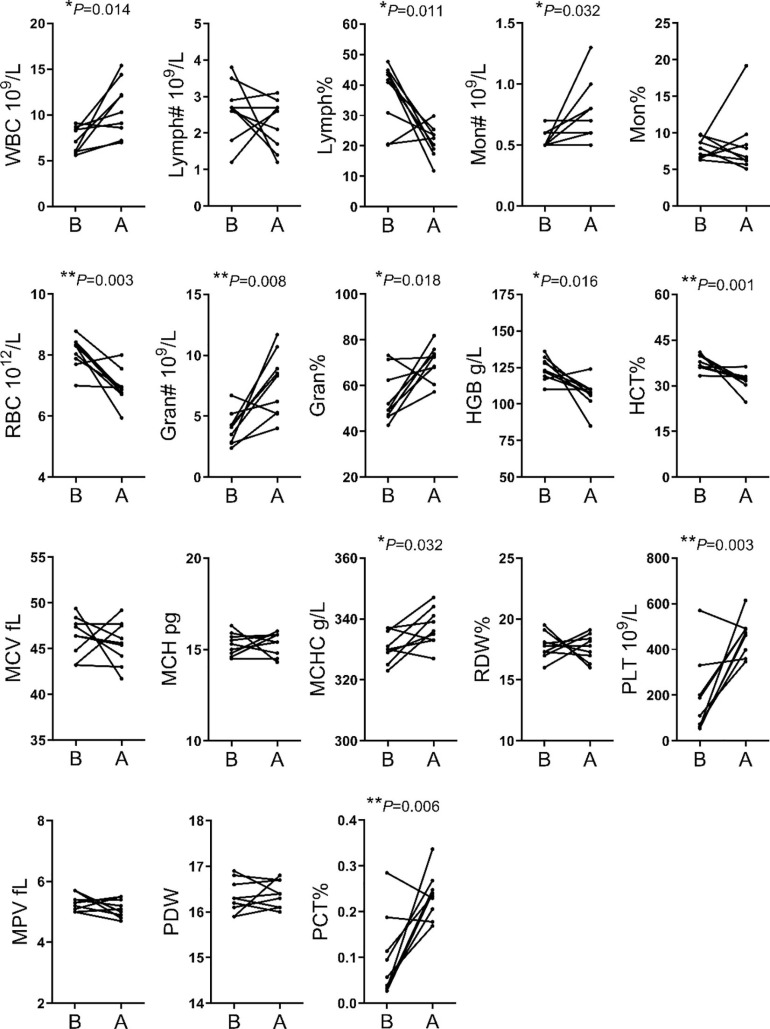
Comparison of hematological indices between before and after transportation. B, before transportation; A, after transportation; WBC, white blood cell count; LYMPH#, lymphocyte number; LYMPH%, lymphocyte percentage; MON#, monocyte number; MON%, monocyte percentage; RBC, red blood cell count; Gran#, neutrophil granulocyte number; Gran%, neutrophil granulocyte percentage; HGB, hemoglobin; HCT%, hematocrit; MCV, average red blood cell volume; MCH, average red blood cell hemoglobin amount; MCHC, average red blood cell hemoglobin concentration; RDW, red blood cell percentage; PLT, platelet number; MPV, average platelet volume; PDW, platelet distribution width; PCT%, thrombocytocrit.

The analysis of hematological indices showed significant changes in 11 of the 18 indices evaluated. The WBC, MON#, Gran#, Gran%, MCHC, PLT and PCT% were higher after transportation compare to before transportation. The RBC, HGB, LYMPH% and HCT% were lower after transportation compare to before transportation (Figure 1).

**FIGURE 2:**
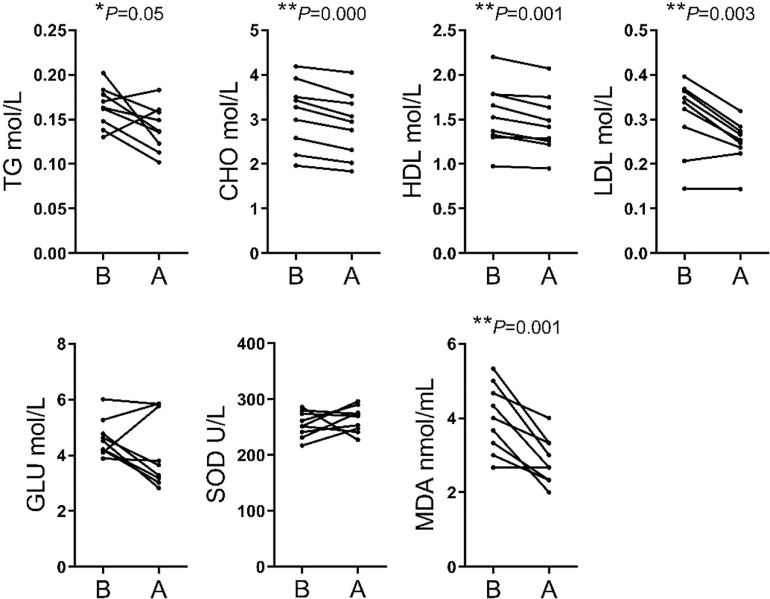
Comparison of serum biochemical parameters between before and after transport. TG, triglyceride; CHO, cholestenone; HDL, high-density lipoprotein; LDL, low-density lipoprotein; GLU, glucose; SOD, superoxide dismutase; MDA, malondialdehyde.

The analysis of biochemical parameters showed significant differences in 5 of the 7 indices assessed. The TG, CHO, HDL, LDL, and MDA contents were lower after transportation compare before transportation, whereas GLU and SOD levels did not differ significantly between the two stages (Figure 2).

### RNA-Seq Data and Verification by qPCR

**FIGURE 3:**
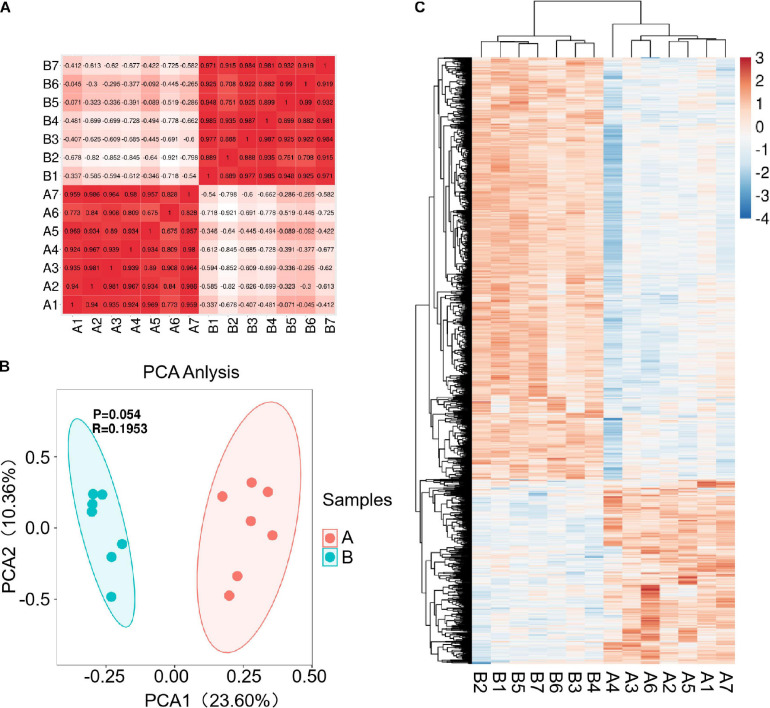
Correlation and differentially expressed genes (DEGs) before and after transportation. **(A)** Correlation between the 14 samples. **(B)** PCA analysis between 14 samples. **(C)** Heat map of DEGs (FC > 2 and FDR < 0.05). B, before transportation; A, after transportation; FC, fold change; TPM, transcripts per million.

**FIGURE 4:**
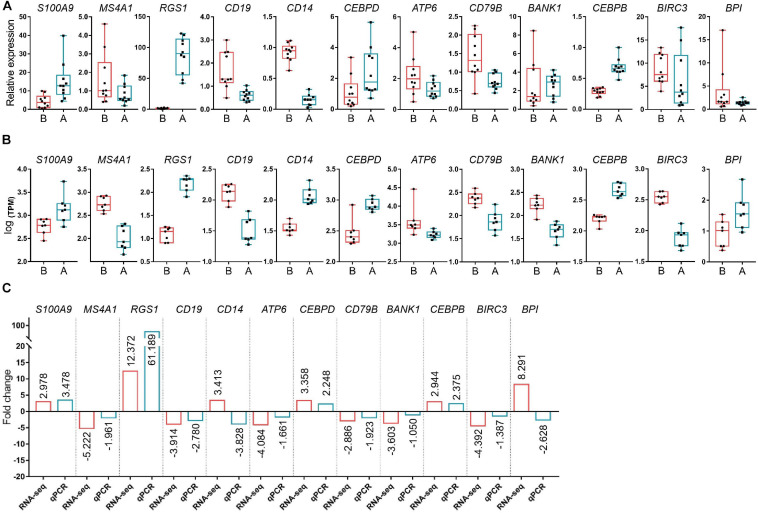
Verification of the differentially expressed genes (DEGs) by PCR. **(A)** The relative expression levels of the selected DEGs by qPCR. **(B)** Log [transcript count per million (TPM)] of DEGs by RNA-seq. **(C)** Fold-change of DEG levels for both Log (TPM) and qPCR. *S100A9, S100 calcium binding protein A9; MS4A1, membrane spanning 4-domains A1; RGS1, regulator of G protein signaling 1; CD19, CD19 molecule; CD14, CD14 molecule; CEBPD, CCAAT enhancer binding protein delta; ATP6, ATP synthase F0 subunit 6; CD79B, CD79b molecule; BANK1, B cell scaffold protein with ankyrin repeats 1; CEBPB, CCAAT enhancer binding protein beta; BIRC3, baculoviral IAP repeat containing 3; BPI, bactericidal permeability increasing protein; ACTB, actin beta*.

Fourteen libraries representing seven animals in each group (before and after transportation) were prepared from total leukocyte mRNA, and group B was the reference group. The range of reads count for the 14 samples was 34888216∼43650486. For each sample, the number of total mapped reads was >90%, the Q20 base ratio was >97%, and the Q30 base ratio was >92% (Table 2). Correlation analysis showed that 14 samples were classified into two groups (group B and group A; Figure 3A). The result of principal component analysis (PCA) was same as correlation analysis, 14 samples also were divided into two groups (group A and group B; Figures 3B,C). There were 1,092 DEGs with FDR <0.05 and fold change >2 as the cut-off criterion, including 329 up-regulated and 763 down-regulated genes (Supplementary Material 1). Twelve DEGs (fold-change >2 and FDR <0.05) were randomly chosen for verification by qPCR. Nine genes showed the same trend as the RNA-seq data (*S100A9*, *MS4A1*, *RGS1*, *CD19*, *CEBPD*, *ATP6*, *CD79B*, *CEBPB*, and *BIRC3*), two genes showed the opposite trend (*CD14* and *BPI*) and another gene was not significantly different between before and after transportation by qPCR (*BANK1*). The consistency of verification was 75% (Figure 4).

**TABLE 2:** Quality control summary of RNA-seq data.

Samples	Total reads	Total mapped	Uniquely mapped	Q20 bases ratio (%)	Q30 bases ratio (%)
B1	38,462,826	37,100,100 (96.46%)	36,216,024 (94.16%)	98.23	94.06
B2	39,975,884	38,224,770 (95.62%)	37,372,975 (93.49%)	97.92	93.26
B3	38,191,018	37,083,200 (97.10%)	36,289,115 (95.02%)	98.37	94.32
B4	43,252,526	41,525,896 (96.01%)	40,503,909 (93.65%)	98.13	93.80
B5	39,930,818	37,845,749 (94.78%)	36,913,114 (92.44%)	98.13	93.76
B6	43,368,036	41,935,855 (96.70%)	40,851,175 (94.20%)	98.16	93.88
B7	37,401,922	35,636,281 (95.28%)	34,743,232 (92.89%)	97.70	92.75
A1	41,095,894	39,538,316 (96.21%)	38,450,721 (93.56%)	98.19	93.98
A2	38,588,200	36,822,949 (95.43%)	35,764,936 (92.68%)	97.89	93.25
A3	34,888,216	33,538,260 (96.13%)	32,587,209 (93.40%)	98.26	94.17
A4	43,261,498	41,218,974 (95.28%)	39,843,128 (92.10%)	98.26	94.18
A5	39,960,690	38,037,925 (95.19%)	36,829,222 (92.16%)	98.32	94.30
A6	43,650,486	41,891,496 (95.97%)	40,613,669 (93.04%)	97.94	93.35
A7	39,184,496	37,485,708 (95.66%)	36,382,749 (92.85%)	98.16	93.93

*B, before transportation; A, after transportation.*

### Enrichment Analysis of DEGs Between Before Transport and After Transport

**FIGURE 5:**
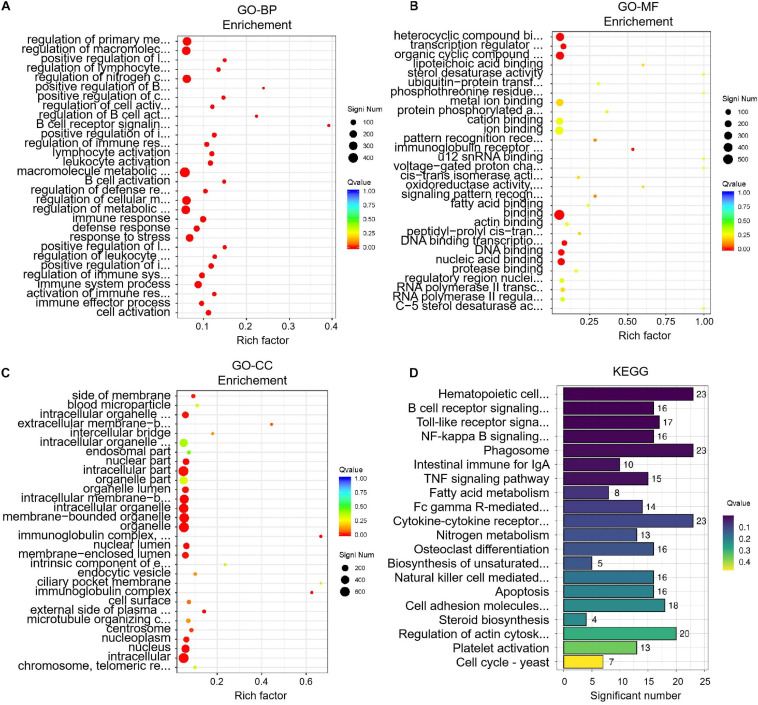
Enrichment analysis of differentially expressed genes (DEGs) before and after transportation. **(A)** GO-BP analysis. **(B)** GO-MF analysis. **(C)** GO-CC analysis. **(D)** KEGG analysis.

To identify key differences between before and after transportation, GO and KEGG enrichment were performed to determine DEGs’ function. The biological process (BP) of GO terms showed significant genes those are involved in cell activation, immune effector process, activation of the immune response, immune system process, regulation of immune system process, etc (Figure 5). The molecular function (MF) of GO terms shows the protease binding, regulatory region nucleic acid binding, RNA polymerase II transcription factor complex, RNA polymerase II regulatory region sequence-specific DNA binding, C-5 sterol desaturase activity, etc (Figure 5). The most enriched GO terms in cellular component (CC) were chromosome/telomeric region, nucleus, intracellular, nucleoplasm, centrosome, etc. (Figure 5). The KEGG analysis showed 200 pathways were significantly enriched. The pathways were mainly cell cycle-yeast, platelet activation, fatty acid metabolism, steroid biosynthesis, and intestinal immune for IgA. The KEGG analysis results showed that the DEGs were mostly associated with the immune response, lipid metabolism, hormone regulation, etc (Figure 5). The other pathways have been presented in Supplementary Materials 2, 3.

### Co-expression Analysis, Key Module, and Hub Genes Identification

**FIGURE 6:**
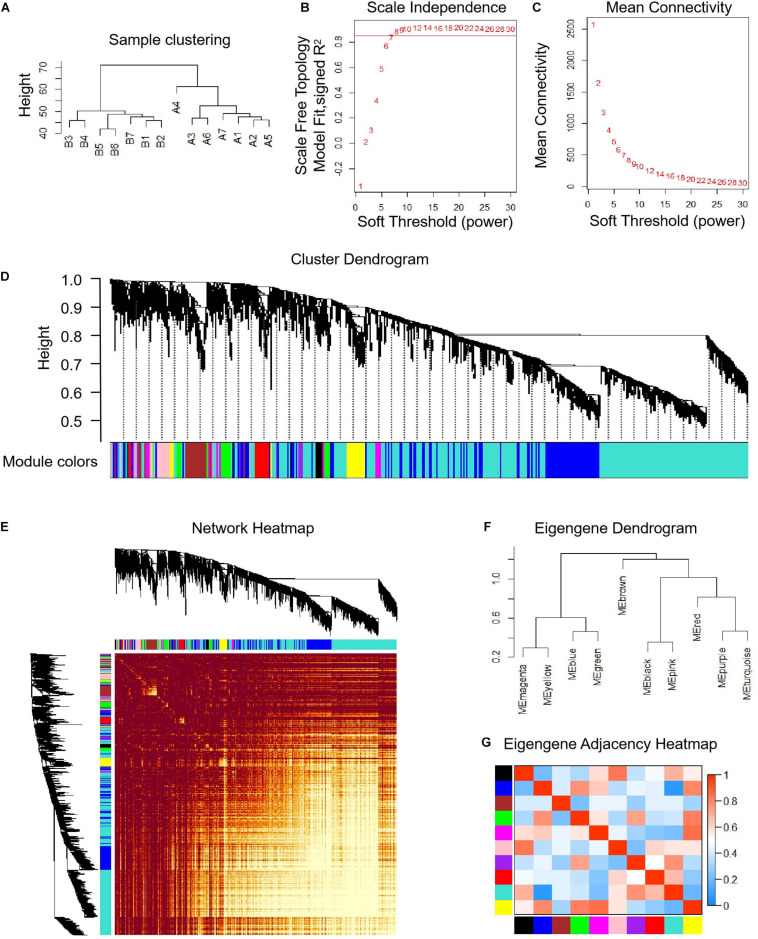
Co-expression analysis of differentially expressed genes (DEGs) before and after transportation. **(A)** Samples clustering analysis of before and after transportation. **(B)** Scale independence of co-expression. **(C)** Mean connectivity of co-expression. **(D)** Cluster dendrogram of DEGs. **(E)** Network heatmap plot of DEGs. **(F)** Eigengene dendrogram of modules. **(G)** Eigengene adjacency heatmap.

**FIGURE 7:**
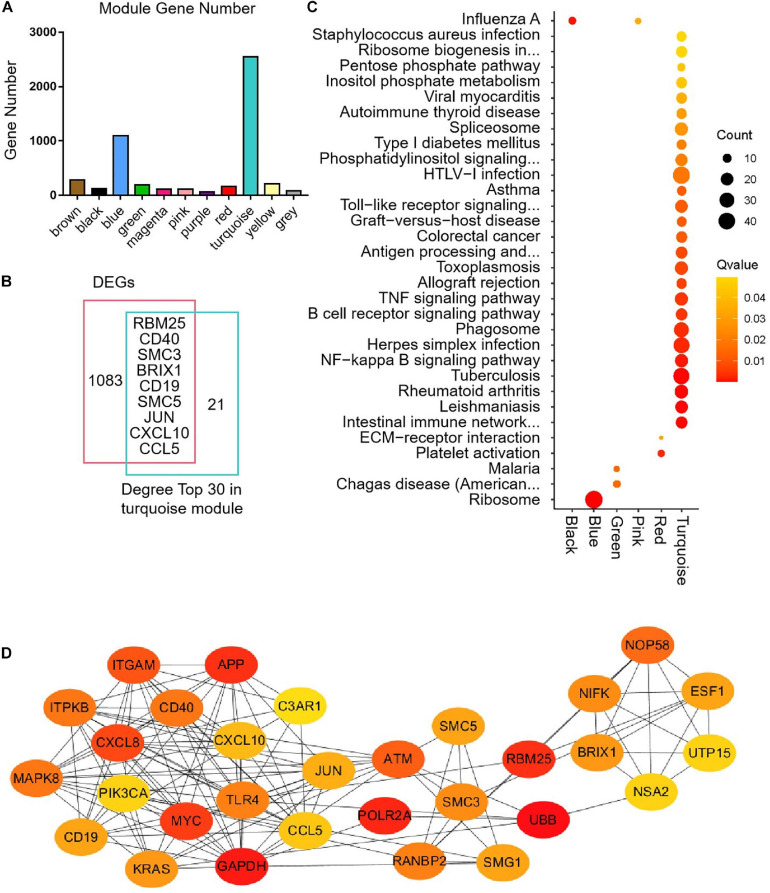
Modules gene enrichment and protein-protein interaction (PPI) network analysis. **(A)** The number of genes in the different clusters. **(B)** Venn analysis between DEGs and degree top 30. **(C)** KEGG enrichment of different modules. **(D)** PPI network of the hub genes in turquoise module.

**FIGURE 8:**
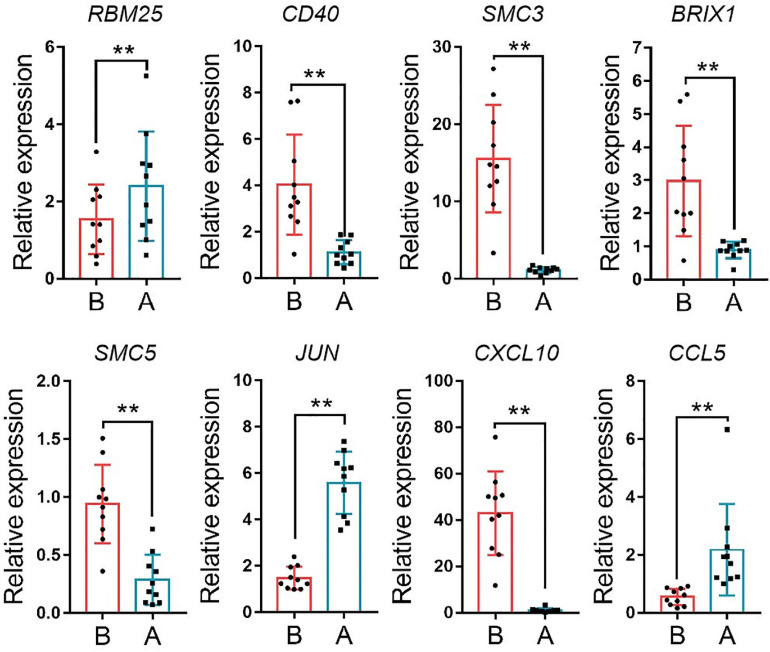
The expression of hub gene in turquoise module. *RBM25, RNA binding motif protein 25, CD40, CD40 molecule, SMC3, structural maintenance of chromosomes 3, BRIX1, biogenesis of ribosomes BRX1, SMC5, structural maintenance of chromosomes 5, JUN, jun proto-oncogene, CXCL10, C-X-C motif chemokine ligand 10; CCL5, C-C motif chemokine ligand 5*. **P < 0.01.

The median absolute deviation (MAD) top 5,000 genes were used to construct a co-expression network (Supplementary Material 4). 14 samples were clustered in two branches (Figure 6A). According to the results of scale independence and mean connectivity, we took power as 8 when correlation index reached 0.85 (Figures 6B,C). All the genes were clustered into eleven modules (Figure 6D). The modules genes see Supplementary Material 5. The results of network heatmap indicated a significant difference among modules (Figure 6E). The most extensive module was the turquoise module, containing 2,546 genes. The smallest module was purple module, containing 68 genes (Figure 7A). After KEGG enrichment in different modules (Supplementary Materials 6, 7), the turquoise module was the most essential module associated with short distance transportation stress, including immune reaction and metabolic pathway (Figure 7C). Then PPI protein network of turquoise was constructed in STRING (Supplementary Material 8). The Cytohubba plugin based on Cytoscape was used to perform the PPI network analysis (Supplementary Material 9) and the top 30 genes based on degree algorithm were identified as potential hub genes (Figure 7D), which contains 9 DEGs (Figure 7B). Considering fold change of qPCR was more comfortable to detect in practical production and the gene function of B cell receptor signal pathway, *structural maintenance of chromosomes 3* (*SMC3*), *jun proto-oncogene* (*JUN*) and *C-X-C motif chemokine ligand 10* (*CXCL10*) could be considered as molecular markers in short-distance transportation stress (Figure 8).

## Discussion

Beef cattle transportation is an inseparable part of the modern beef cattle industry, and since 2 decades, both transnational and cross-regional transportation were increased significantly. The neglect of beef cattle transportation stress by producers renders it difficult to accurately estimate the beef cattle industry (Deters and Hansen, 2020). It has been estimated that the death rate due to beef cattle transportation is in the range of 1–14%. Transportation stress is multifaceted but can be divided into long distance and short distance transportation stress (Marti et al., 2017). Long distance transportation stress can lead to a decline in the body’s resistance, as well as a comprehensive, pathological, whole-body reaction syndrome (such as fever, depression, loss of appetite, thick coat, cough, asthma, sticky or purulent nasal fluid, diarrhea, bloody stools, arthritis, conjunctivitis, and even death) (Padalino et al., 2017; Miranda-de la Lama et al., 2018). Comparing with long distance transportation stressors, short distance transportation stressors were less, mainly include fear, cold or heat, and lack of food and water (Allano et al., 2016).

Additionally, less attention has been paid to short distance transportation stress as it does not result in obvious economic losses, and most of the symptoms disappear automatically (Simova et al., 2017). In rare cases, post-stress symptoms can occur, and drug intervention is needed (Van Engen et al., 2016; Capik et al., 2017; Wu et al., 2020). Mitigating beef cattle transportation stress mostly relies on experience and lacks a theoretical scientific basis. Consequently, the relevant underlying molecular mechanisms must be determined to alleviate transportation stress. In this study, we constructed a short distance transportation stress model to obtain the biological blood information and identify the molecular mechanism related to the TSSBC, thereby providing a reference for alleviating this condition. We collected the venous blood of beef cattle before and after transportation, and subsequently measured the blood hematological and biochemical indices, and analyzed the blood transcriptome.

Numerous indices were recorded in hematological parameters. The increases of MON#, Gran#, Gran%, MCHC, PLT, and PCT% were related to enhanced immune response following external stimulation. The increases of WBC was an essential phenotype in transportation stress (Dalin et al., 1993; Endo et al., 2017). Meanwhile, the decreases of RBC, HGB, and HCT% may simply have resulted from irregular drinking and eating before and after transportation. Finally, the high WBC and PLT were related to handling, loading, traveling and intense physical activity during loading (Pascual-Alonso et al., 2017). We found that the TG, CHO, HDL and LDL contents were significantly decreased for blood biochemistry parameters. This might be related to the fasting and lack of drinking water during transportation. Short term fasting could promote energy uptake by tissues and organs, but the time of fasting in this study could not enough for glycogenolysis and the following lipidolysis. Besides, the level of MDA showed that cattle have increased resistance to oxidative stress (Huang et al., 2000). SOD level was insignificant between group B and group A, but had an upward trend. It showed that most cattle improved ability to scavenge oxygen free radicals (Weismann et al., 2011).

WBC were the only nucleated cell in the blood, and were used to compare the gene expression between two groups by RNA-seq. We first evaluated the quality of the sequencing data, and found that the number of total mapped reads was >95%, the Q20 base ratio was >97%, and the Q30 base ratio was >92% for each sample, indicating that the quality of RNA sequencing data was reliable and could be used for further investigation. Next, we identified DEGs based on the sequencing data by DESeq2. To verify the accuracy of the sequencing data, we randomly selected 12 DEGs for qPCR and found that the results had 75% accuracy, which implied that the data were suitable for further analysis.

We performed GO and KEGG enrichment analysis of DEGs. Combining with the BP enrichment results top 20 terms showed that the level of humoral immunity with B cells as the core decreased after transportation (Honda et al., 2015). The decrease of humoral immunity explained that cattle were more vulnerable to disease after transportation (Quinteiro-Filho et al., 2010, 2012; Calefi et al., 2014; Gomes et al., 2014). It was suggested that good management should be provided after transportation to help cattle recover from transportation stress. Like BP enrichment, KEGG enrichment results showed that B cell receptor function had a significant change after transportation. Besides, fatty acid metabolism and steroid biosynthesis were enriched between two groups, which was same as the results of the changing of TG, CHO, HDL, LDL in blood after transportation, and showed that hypothalamus-endocrine organ axis could be the core of transportation stress regulation. We hypothesized that B cell dysfunction might be the hematological molecular mechanism of short-distance transport stress, and the decrease of humoral immunity could be the potential factor behind the economic loss.

**FIGURE 9:**
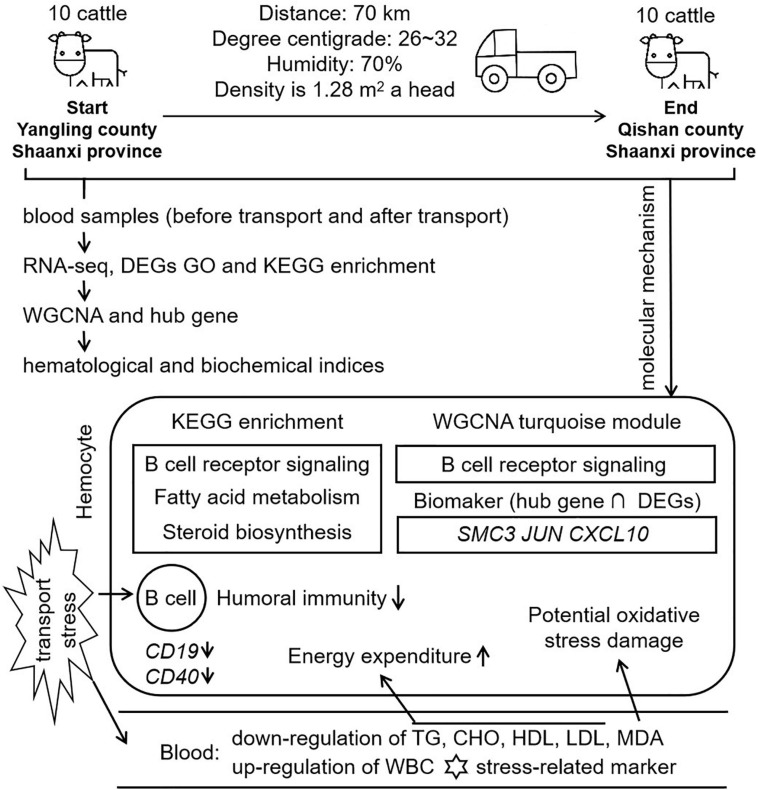
Workflow and molecular mechanism in this study. In this study, we constructed a short distance transportation stress model of beef cattle. Blood samples were collected and performed to RNA-seq, GO, KEGG, and WGCNA. Comparing with hematological and biochemical indices, we concluded that B cell function, lipid metabolism and steroid function were the mean molecular mechanism in short distance transportation stress of beef cattle. *CD19* and *CD40*, the markers of B cell, were down-regulated in transcriptome data. The results of WGCNA showed that B cell function could be the core of transportation stress. After hub gene identification and qPCR verification, *SMC3*, *JUN*, and *CXCL10* could be suggested as potential biomarkers for the diagnosis and recover of this condition.

The main objective of this study was utilize a global approach to construct a gene co-expression network that could predict clusters of candidate genes involved in transportation stress. We hypothesized that tightly co-expressed gene modules, enriched in shared functional annotation, would provide the most effective predictions of candidate gene sets that might underlie a given biological process. Here, WGCNA was applied to investigate MAD Top 5,000 genes among the 14 samples in this study, and constructed 11 independent modules. We applied KEGG to evaluate the function of genes in different modules. Finally, turquoise module was enriched in metabolic pathway and immune reaction related to transportation stress. The pathway enriched in other modules related to transportation stress had no direct connection with transportation stress. It is reminding that housing disinfection and more attention to cattle’s health were effective methods for reducing losses even short distance transportation. All the genes in turquoise module were used to construct PPI network, and degree top 30 genes were identified as hub genes by Cytohubba plugin based on Cytoscape. *CD19* and *CD40* were two specific B cell surface markers and have an important essential function of B cell maturation. The other hub genes were associated with the function of immune, differentiation, proliferation, survival, and apoptosis. Combing with the results of GO and KEGG enrichment, the function of hub genes was centered around B cell function (Hu et al., 2018; Gazon et al., 2018; Karin and Razon, 2018; Tokunaga et al., 2018; Xiao et al., 2019; Huang et al., 2020; Yang et al., 2020). Considering fold change of qPCR was easier to detect in practical production and the gene function of B cell receptor signal pathway, *SMC3*, *JUN* and *CXCL10* could be suggested as molecular markers in transportation stress. *SMC3*, important member in cell cycle pathway, could be the regulator in the differentiation and activation of B cell function (Hu et al., 2018). *JUN* was belonged to many pathways related to B cell function, and had other functions containing differentiation, proliferation, survival, and apoptosis (Gazon et al., 2018; Figure 9). As a critical factor in immune activation through paracrine signaling, the decreasing of *CXCL10* showed that immune cell differentiation was weakened, and the cattle susceptible to infection by germs (Karin and Razon, 2018; Tokunaga et al., 2018).

For the first time of this study, WGCNA was used to identify co-expression gene sets associated with short distance transportation stress. Turquoise module was found to be highly enriched for genes involved in the immune response and metabolism pathway. Collectively, the blood RNA-seq analysis and WGCNA indicated that the changing of B cell differentiation, proliferation, survival, and apoptosis were the potential threats in short distance transportation stress. Besides, *SMC3*, *JUN*, and *CXCL10* were three potential molecular markers for short distance transportation diagnosis and recover.

## Data Availability Statement

The high-throughput sequencing data of the original RNA-seq has been saved in the NCBI Sequence Reading Archive (https://www.ncbi.nlm.nih.gov/sra/), with the accession number PRJNA658391 (SRR12800407–SRR12800420).

## Ethics Statement

The animal study was reviewed and approved by Institutional Animal Care and Use Committee of Northwest A&F University.

## Author Contributions

HZ, MW, and SW analyzed the data. HZ, XT, XY, and SL wrote the manuscript. HZ, XT, MW, QL, XY, SL, and JJ collected the samples. QL performed the qPCR. XY and SL reviewed and edited the manuscript. XS designed the experiment. All authors contributed to the interpretation of the results and writing of the manuscript.

## Conflict of Interest

The authors declare that the research was conducted in the absence of any commercial or financial relationships that could be construed as a potential conflict of interest.

## Footnotes

1 http://www.bioinformatics.babraham.ac.uk/projects/fastqc/

2 http://www.usadellab.org/cms/?page=trimmomatic

3 https://daehwankimlab.github.io/hisat2/

4 https://www.kegg.jp/

5 http://david.abcc.ncifcrf.gov/

6 https://string-db.org/

## Supplementary Material

Supplementary Material 1 DEGs list.

Supplementary Material 2 DEGs GO enrichment.

Supplementary Material 3 DEGs KEGG enrichment.

Supplementary Material 4 Mad TOP 5000 gene list.

Supplementary Material 5 Modules genes list.

Supplementary Material 6 Module gene GO enrichment.

Supplementary Material 7 Module gene KEGG enrichment.

Supplementary Material 8 Turquoise module string interactions.

Supplementary Material 9 Turquoise module cytphubba degree.
